# Treatment of Transvestic Fetishism With Fluoxetine: A Case Report

**Published:** 2012

**Authors:** Mohammad Amir Usmani, Rakesh K Gaur, Suhail Ahmad Azmi, Sheshank Gangwar

**Affiliations:** 1 Department of Psychiatry, J.N. Medical College, A.M.U. Aligarh, U.P., INDIA.

**Keywords:** Fluoxetine, Paraphilia, Transvestic Fetishism

## Abstract

Transvestic fetishism is having a sexual or erotic interest in cross-dressing. This case report describes a 17-year-old male with transvestic fetishism who suffered from obsessive thoughts and subsequent masturbation as compulsion. He was managed successfully with fluoxetine.

## Introduction

Transvestic fetishism is having a sexual or erotic interest in cross-dressing. It differs from cross-dressing for entertainment or other purposes that do not involve sexual arousal and is categorized as a paraphilia in the Diagnostic and Statistical Manual of the American Psychiatric Association. There are two key criteria before a psychiatric diagnosis of "transvestic fetishism" is made(1):recurrent, intense sexually arousing fantasies, urges, or behavior, involving cross-dressing.This causes clinically significant distress or impairment, whether socially, at work, or elsewhere.

## Case report

A 17-year-old Indian male presented to the psychiatry outpatient department with the complaint of having recurrent desires to wear the clothes of his mother. The thought was very distressing to the patient andcame to his mind again and again until he wore those clothes and masturbated in it. For this purpose, he stole a complete set of his mother’s clothes. He has been wearing the clothes secretly for the past 2 years solely for the purpose of pleasure. Recently he was caught by the parents while wearing the clothes and masturbating and then beaten by the parents. On further enquiry he admitted that the thought is obsessive in nature, he tries to control it but is not able to do so. He added that he experiences restlessness until he wears the clothes and masturbates in order to overcome the anxiety. The thought caused significant impairment in his studies and other social functioning. He did not have any desire to be treated as female nor did he have any preoccupations regarding hormonal or surgical treatment for sex change therapy.There was a past history of homo-sexual activity but upon presentation he denied having any sexual relationship. His general physical and neurological examinations were within normal limits. On investigations, contrast-enhanced CT scan of the brain and electroencephalograph were also found to be normal.

He was diagnosed provisionally as a case of transvestic fetishism and fluoxetine (20 mg once a day) was prescribed. The dose of fluoxetine was increased to 40 mg once a day after two weeks. The patient was followed for the next 6 weeks. He showed significant improvement with adecrease in recurrent thoughts and urge to masturbate.

## Discussion

The case described above was not having the desire to be treated as female. He was not preoccupied with any thought of sex change. The problem which he suffered was obsessive thoughts of wearing female clothes and subsequent compulsive masturbation. The psychopathology leads to the obsessive spectrum of the disease rather than gender identity disorder. However, transsexualism and erotic arousal to cross-dressing are not mutually exclusive (2).

There is no established drug for treatment of transvestic fetishism. Stein et al. described paraphilias as a neurologiacal spectrum disorder and considered obsession and compulsion at the compulsive end and paraphilias at the impulsive end of the spectrum. He also discussed the use of serotonergic medications for the treatment of sexual obsessions (3). Fedoroff reported successful treatment of transvestic fetishism with buspirone(4). Jogerson also reported successful treatment of cross dressing with fluoxetine(5).Use of sertraline and lithium (6) as well as escitalopram(7)has also been reported for this disorder. Abdo et al. have reported two cases in which transvestism and obsessive-compulsive disorder (OCD) were related and suggested that the case with more ego dystonic thoughts was more responsive to treatment(8).

The case report highlights the possible association of paraphilias and obsession. Administration of fluoxetine would be useful for management of an OCD-transvestism continuum disorder.

## Authors’ Contributions

MAU designed the evaluation and drafted the manuscript. RKG and SAA reevaluated the diagnosis and revised the manuscript. ShG helped in acquisition of clinical data and editing the manuscript. All authors read and approved the final manuscript.

## Notes

**Declaration of interest:** None.
